# Problematic Internet Use, health behaviors, depression and eating disorders: a cross-sectional study among Polish medical school students

**DOI:** 10.1186/s12991-022-00384-4

**Published:** 2022-02-11

**Authors:** Marta Kożybska, Jacek Kurpisz, Iwona Radlińska, Edyta Skwirczyńska, Natalia Serwin, Paulina Zabielska, Artur Kotwas, Beata Karakiewicz, Zofia Lebiecka, Jerzy Samochowiec, Kinga Flaga-Gieruszyńska

**Affiliations:** 1 Subdepartment of Medical Law, Department of Social Medicine, Pomeranian Medical University in Szczecin, Szczecin, Poland; 2 Department and Clinic of Psychiatry, Pomeranian Medical University in Szczecin, Szczecin, Poland; 3 Department of Medical History and Ethics, Pomeranian Medical University in Szczecin, Szczecin, Poland; 4 Department of Laboratory Medicine, Pomeranian Medical University in Szczecin, Szczecin, Poland; 5 Subdepartment of Social Medicine and Public Health, Department of Social Medicine, Pomeranian Medical University in Szczecin, Szczecin, Poland; 6 Research Team On the Civil Procedural Law and the Informatisation of the Judiciary, Faculty of Law and Administration, University of Szczecin, Szczecin, Poland

**Keywords:** Problematic Internet Use students, Poland, Health behaviors, Depression, Eating disorders

## Abstract

**Background:**

Problematic Internet Use is defined as a use of the Internet which leads to various difficulties. The aim of this study was to check whether Problematic Internet Use is associated with health risks, such as: anti-health behaviors, depressive symptoms, abnormal body weight or eating disorders.

**Methods:**

This cross-sectional study included 540 medical school students of Polish descent (83.5% females; 16.5% males), whose mean age was 22.49 years (SD = 5.20). The participants were asked to complete a questionnaire set, including the Problematic Internet Use Test, Juczyński’s Health-Related Behavior Inventory, the Beck Depression Inventory, the Eating Attitudes Test and a self-designed demographic survey.

**Results:**

Increased Problematic Internet Use scores were observed in male, full-time students, persons who use the Internet on the computer (compared to those who mostly use it on the phone), and those who go online mainly for entertainment purposes (compared to those who indicated another main purpose of using the Internet). 47.6% of the sample reported poor health behaviors, while 27.1% met the criteria of a depressive episode and 6.9% of an eating disorder. High risk of Problematic Internet Use was observed in 2.8% of the sample, particularly those who reported having more free time during the day, engaged in fewer health protective behaviors, manifested more severe depressive symptoms and scored higher on the Eating Attitudes Test.

**Conclusion:**

Such results indicate that students with Problematic Internet Use lead an unhealthy lifestyle and more often show symptoms of depression and eating disorders than students without Problematic Internet Use.

## Introduction

In the modern world, the Internet constitutes an important tool for work, especially during the SARS-CoV-2 pandemic. It is also used as a common pastime. This can sometimes lead to appearance of behaviors exceeding those that involve its normal use for acquiring knowledge, communication and entertainment purposes. The negative effects of Internet abuse were known before the SARS-CoV-2 pandemic, which is confirmed by the data presented in our article. Problematic Internet Use (PIU) is defined as a use of the Internet which leads to the occurrence of psychological, social, academic and occupational difficulties [1]. Moreno et al. emphasize that the diagnosis of PIU requires consideration of not only the effects of Internet use, but also its specific characteristics. They describe PIU as “*Internet use that is risky, excessive or impulsive in nature leading to adverse life consequences, specifically physical, emotional, social or functional impairment”* [2]*.*

The Interaction of Person-Affect-Cognition-Execution (I-PACE) model lists depression as PIU-related psychopathological factor [3]. Correlations between addictive Internet use and depressive symptoms have been reported in various studies [4–7]. In depressed individuals, Internet use is believed to constitute a strategy of coping with negative emotions [3, 8, 9] or stressful situations [3, 10]. Jelenchick et al. note that depressive symptoms (e.g., anhedonia or impaired concentration) may additionally hinder healthy lifestyle choices, promoting abandoning other daily activities in favor of staying online. Neglecting health behaviors may result in developing symptoms of depression, such as difficulty sleeping or concentrating, low energy levels, or poor appetite [4].

Research suggests associations between individual negative health behaviors and PIU. These include being under- or over-weight [11, 12], sedentary lifestyle, gambling [11], eating fast food [13], abuse of alcohol or other psychoactive substances [14, 15], migraines, back pain, or insufficient rest [12]. To the best of our knowledge, no comprehensive research to date has tackled health behaviors of Internet users that would include both their general description and their specific domains, i.e., eating habits, mental attitude, preventive behaviors and health practices.

The existing reports indicate associations between PIU and eating disorders [12, 16–20], though not all available research seems to confirm such links [19, 21]. Based on the above studies, Canan points out that Internet addiction and abnormal eating habits are closely related [20]. Such concepts are certainly worth expanding on.

A cohort that has been most thoroughly studied in relation to PIU are young adults. On the one hand, they are particularly vulnerable to behavioral addictions [22], on the other, the Internet is an integral part of their lives, being a tool they use for entertainment, developing interests and passions, studying, work or maintaining social contacts. On top of that, to facilitate communication and teaching, universities usually provide and require Internet access [23], e.g., through e-learning platforms, e-mail contact with lecturers, the Dean's office, or online course enrolment. During the SARS-CoV-2 pandemic, e-learning has often become the only teaching option. Students also tend to use longer breaks between classes to browse the Internet [24]. All that, together with moving away from home, the feeling of new-found freedom, independence, and other factors related to early adulthood, is likely to contribute to an increased risk of excessive Internet use [24].

To date, research on the Internet use among young adults included mainly students from Western Europe, the USA, and Asian countries. Considerably fewer scientific reports on the subject come from central Europe, including Croatia [25, 26], Hungary [27] and Poland [28], or Poland and Russia [29].

All things considered, we decided to supplement the existing knowledge base by presenting the results we obtained before the outbreak of the SARS-CoV-2 pandemic. Our aim was to determine the relationship between Problematic Internet Use, health behaviors, BMI, symptoms of depression and eating disorders among students of a medical university in Poland. On the one hand, students of health-related majors should exhibit high empathy levels [30] and openness to interpersonal contact, both of which negatively correlate with PIU, but on the other they are exposed to stress (demanding classes, first contact with patients), which puts them at a higher risk of developing PIU [31].

We hypothesized PIU to be associated with reduced health-promoting behaviors, higher BMI, symptoms of depression and eating disorders. We also expected full-time students who have more free time and use the Internet for entertainment purposes, mainly via their smartphones to be at a greater PIU risk.

## Methods

### Participants and procedure

This cross-sectional study was conducted between October 2017 and February 2018 among students of Pomeranian Medical University in Szczecin (PMU). Upon receiving their informed consent to participate, students completed provided questionnaire sets during classes. The inclusion criterion was a PMU student status. Participation was voluntary, anonymous and free of charge. Participants did not receive any incentives to participate in the study. Students were informed about research objectives, questionnaire completion procedures and the possibility to withdraw at any stage with no consequences. All participants received information on free psychological and psychiatric assistance in the city of Szczecin.

A total of 540 students were recruited to participate in the study, with two excluded due to missing data. Thus, the final sample consisted of 538 participants.

### Measures

The questionnaire set used in the study included: the Problematic Internet Use Test (TPUI22) [32], Zygfryd Juczyński’s Health-Related Behavior Inventory (IZZ) [33], the Beck Depression Inventory (BDI-I) [34], the Polish version of the Eating Attitudes Test (EAT-26) [35], and a self-designed survey.

Problematic Internet Use (PIU) was measured with the Problematic Internet Use Test (TPUI22), which is a modified Polish adaptation of Kimberly Young’s Internet Addiction Test (IAT), developed by Ryszard Poprawa. The score range is 0–110, where a higher score indicates a higher risk of Problematic Internet Use. The score indicates very low (0–1 for persons ≤ 24 years and 0 for persons > 24 years), low (2–10 for persons ≤ 24 years and 1–6 for persons > 24), medium (11–49 for persons ≤ 24 years and 7–41 for persons > 24), high (50–79 for persons ≤ 24 years and 42–75 for persons > 24) or very high risk of Internet addiction (80–110 for persons ≤ 24 years and 76–110 for persons > 24). The tool is characterized by high reliability (Cronbach’s *alpha* = 0.935). In our sample, Cronbach’s alpha was 0.90. It is composed of 22 questions, the answers to which are: never, sporadically, rarely, sometimes, often, and always [32]. For the purposes of this study, PIU scores were used and analyzed to: (a) divide the sample into groups at various risks of Internet addiction (categorized as: very low, low, medium, high, very high); (b) determine the severity of PIU in the entire sample (as a continuous variable).

Health behaviors were assessed using Zygfryd Juczyński’s Health-Related Behavior Inventory (IZZ). The scale contains 24 items concerning health-related behaviors, assessed on a 5-point scale (from 1 = “almost never” to 5 = “almost always”). A higher score means a greater frequency of health behaviors. Raw scores indicate low (24–71 for men and 24–77 for women), medium (72–86 for men and 78–91 for women), or high (87–120 for men and 92–120 for women) intensity of engaging in health behaviors. The tool is also used to identify and assess four subcategories of health behaviors: healthy eating habits, preventive behaviors (avoiding diseases), positive mental attitude and pro-health practices (leading a healthy lifestyle). The questionnaire has excellent psychometric properties. Cronbach’s *alpha* for the entire scale is 0.85, whereas for the four subscales individually it remains in the range from 0.60 to 0.65. In this study, the IZZ scores were used to: (a) distinguish different subsets of the sample based on the intensity of health behaviors (low, medium, high) to enable comparisons; (b) determine the total test score; (c) determine the scores in the individual sub-categories (eating habits, preventive behaviors, positive mental attitude and health practices) [33].

Depressive symptom severity was measured with the Polish version of the Beck Depression Inventory (BDI-I). Cronbach’s *alpha* for this version of the tool is 0.87 [34], while in our sample it was 0.91. In this study, apart from considering participants’ raw scores, we also assigned them to two groups, either meeting (score 11–63) or not meeting criteria for a depressive episode (score 0–10).

Eating disorders were measured with the Polish version of The Eating Attitudes Test (EAT-26) [35]. The questionnaire contains 26 questions assessed on a 5-point scale from “never” to “always”. The total score is in the range from 0 to 78. A score of ≥ 20 indicates a potential risk of an eating disorder and a specialist mental health consultation may be required [36]. Cronbach’s *α *for the questionnaire is 0.84. Factors considered for further analyses included both the total score, as well as meeting (score of 20–78), or not meeting criteria for the risk of developing an eating disorder (score of 0–19).

The self-designed questionnaire included questions about: gender, age, type of studies (full-time/part-time), field and year of studies, other activities (e.g., volunteering, work, caring for a family member), self-assessed financial situation, average amount of free time during the day, purpose of using the Internet, main device for using the Internet (computer, phone/tablet, mixed).

### Statistical analyses

Normality of distributions was examined with the Kolmogorov–Smirnov test. All tested variables were significantly different from the normal distribution (*p *< 0.05). Group comparisons were performed with the use of the Kruskal–Wallis and Mann–Whitney tests. The correlation study was performed using Spearman’s correlation. Statistical significance was set at *p* < 0.05. For statistical analyses, we used SPSS 20 for Windows.

## Results

### Characteristics of the study group

Study sample included mainly female students of nursing and physiotherapy. The mean age of the participants was 22.49 years (SD = 5.20), with 83.5% (n = 449) of females, and 16.5% (*n* = 89) of males. All participants were of Polish origin. Half of the students used their smartphones to connect to the Internet, predominantly to communicate with others. They reported having an average of 2.89 (SD = 2.34) h of free time daily (i.e., time when they did not have to study, work or perform household chores), with a substantial variation among students of different majors (*p* < 0.001). Of all the examined majors, the students of medical analytics reported having the most, while students of nursing the least free time. Table 1 presents the most important descriptive statistics.

**Table 1:** Socio-demographics, Internet use characteristics and PIU, IZZ, BDI, EAT-26 scores

	*n*	%	Mean	SD	Me	Q3–Q1
Gender						
Females	449	83.5				
Males	89	16.5				
Field of study						
Nursing	163	30.3				
Physiotherapy	162	30.1				
Medical analytics	36	6.7				
Cosmetology	32	5.9				
Health psychology	32	5.9				
Medical biotechnology	30	5.6				
Emergency medical services	29	5.4				
Dietetics	25	4.6				
Obstetrics	22	4.1				
Administration and management of health care	7	1.3				
Main device						
Smartphone	275	51.1				
Computer and smartphone comparably	201	37.4				
Computer	62	11.5				
Main purpose of using the Internet						
Communication with others	200	37.2				
Entertainment	106	19.7				
Studying	78	14.5				
Social media	70	13.0				
Other	10	1.9				
Work	9	1.7				
No response or no indication of one main objective	65	12.1				
PIU						
Overall score			17.01	13.24	13	16
Very low	20	3.7				
Low	196	36.4				
Medium	307	57.1				
High	15	2.8				
Very high	0	0				
IZZ						
Overall score			76.15	14.07	77	20.75
Low	256	47.6				
Medium	191	35.5				
High	91	16.9				
IZZ domain						
Healthy eating habits			3.1	0.83	3.17	1.17
Preventive behaviors			3.16	0.81	3.17	1
Positive psychological attitude			3.41	0.77	3.5	1
Pro-health practices			3.02	0.71	3	0.83
BDI						
10 points	146	27.1	8.81	8.21	6	9
EAT-26						
20 points	37	6.9	7.83	6.68	6	7

*PIU* Problematic Internet Use Test, *IZZ* Health-Related Behavior Inventory, *BDI* Beck Depression Inventory, *EAT-26* The Eating Attitudes Test

### Frequency of Problematic Internet Use (PIU), health behaviors, depression and eating disorders

The PIU risk level is defined via assignment to a particular risk group (very low, low, medium, high, very high; categorical variable). As shown in Table 1, high risk of PIU affected 2.8% of the subjects (*n* = 15). A significant number of students reported low intensity of health behaviors (256; 47.6%). 27.1% of the subjects (*n* = 146) met the criteria of a depressive episode and 6.9% (*n* = 37) of an eating disorder.

### Socio-demographics, Internet use characteristics and PIU scores

The Mann–Whitney test indicated significant differences between males and females *(p* < *0.05)* in terms of the overall PIU score (continuous variable), with males scoring higher than females (Table 2).

**Table 2:** Comparison of the groups in terms of the PIU score (continuous variable)

Variables	*df*	χ^2^ and Z scores	*p *value
Sex^a^		*z* = –2.52^a^	**< *0.05***
Study mode (full time/extramural)^a^		*z* = –2.98^a^	**< *0.01***
Study cycle (bachelor, master, uniform)^b^	2	χ^2^ = 8.65^b^	**< *0.05***
Year of studies^b^	3	χ^2^ = 4.09^b^	NS^f^
Field of studies^b^	9	χ^2^ = 32.44^b^	**< *0.001***
Preferred device (computer/smartphone/computer and smartphone comparably)^b^	2	χ^2^ = 11.34^b^	**< *0.01***
Purpose of Internet use^b^	5	χ^2^ = 29.35^b^	**< *0.001***
Severity of IZZ^b, c^	2	χ^2^ = 7.26^b^	**< *0.05***
BDI risk category^a, d^		*z* = –5.01^a^	**< *0.001***
EAT risk category^a, e^		*z* = –1.17^a^	NS

^a^Mann–Whitney *U* test

^b^Kruskal–Wallis test

^c^IZZ severity—groups of low, medium and high frequency of health-related behaviors

^d^BDI risk category—comparison of groups which meet and do not meet diagnostic criteria for a depressive episode

^e^EAT risk category—comparison of groups which meet and do not meet diagnostic criteria for eating disorders

^f^not statistically significant

Apart from gender, also the mode of study (full-time/extramural) significantly differentiated the subjects in terms of their PIU score (continuous variable) (Table 2). Namely, full-time students seemed to be at a higher risk of PIU relative to their extramural counterparts. Single-cycle master degree students achieved the highest PIU scores, followed by first cycle students, with the second cycle students achieving the lowest scores. The applied post hoc tests (Mann–Whitney) revealed that second cycle students scored significantly lower on PIU than those selecting uniform studies.

An important variable to differentiate PIU risk (continuous variable) turned out to be the selected major *(p* < *0.001)* (Table 2)*.* Nursing students scored significantly lower on PIU compared to medical biotechnology, physiotherapy, medical analytics, obstetrics, dietetics, emergency medical services, cosmetology and health psychology students. We found no other relationships between the investigated majors.

The choice of the device to connect to the Internet significantly differentiated PIU scores *(p* < *0.01)* (continuous variable) (Table 2)*.* Students who accessed the Web mainly via a computer or both a computer and a smartphone had higher PIU scores than those who chose to go online mainly on a smartphone.

The primary purpose for using the Internet also significantly affected PIU overall scores (continuous variable) (Table 2). Entertainment, communication with others, and social media were related to higher PIU scores compared to academic purposes; the use for work was associated with lower PIU scores relative to entertainment and social media; and the use for entertainment was linked to higher PIU scores than for communication with others.

The correlation analysis showed significant albeit weak associations between PIU scores (continuous variable) and leisure time (Table 3). Students at a very low PIU risk (categorical variable) had the least free time during the day, while a high PIU risk was linked to having the most free time *(p* < *0.001)* (Table 4).

**Table 3:** Correlations between PIU scores (continuous variable) and other investigated variables

		PIU	Age	Leisure time	BMI	EAT	BDI	IZZ
PIU	Spearman Correlation	1	−0.071	**0.182**^**a**^	−0**.094**^**b**^	**0.133**^**a**^	**0.325**^**a**^	−0**.147**^**a**^
	Significance (bilateral)		0.104	0.000	0.03	0.002	0.000	0.001
	N	538	529	534	538	538	538	538
Age	Spearman Correlation	−0**.071**^**a**^	1	0.100	0.085	−0.054	−0.065	0.074
	Significance (bilateral)	0.104		0.022	0.052	0.216	0.133	0.088
	N	529	529	525	529	529	529	529
Leisure time	Spearman Correlation	**0.182**^**a**^	−**0.100**^**b**^	1	0.023	−0.047	−**0.125**^**b**^	**0.169**^**a**^
	Significance (bilateral)	0.000	0.022		0.602	0.283	0.004	0.000
	N	534	525	534	534	534	534	534
BMI	Spearman Correlation	−**0.094**^**b**^	0.085	0.023	1	**0.085**^**b**^	0.001	−0.017
	significance (bilateral)	0.03	0.052	0.602		0.049	0.988	0.694
	N	538	529	534	538	538	538	538
EAT-26	Spearman Correlation	**0.133**^**a**^	–0.054	−0.047	0.085	1	**0.376**^**a**^	−0.031
	significance (bilateral)	0.002	0.216	0.283	0.049		0.000	0.473
	N	538	529	534	538	538	538	538
BDI	Spearman Correlation	**0.325**^**a**^	–0.065	−**0.125**^**b**^	0.001	**0.376**^**a**^	1	−**0.441**^**a**^
	significance (bilateral)	0.000	0.133	0.004	0.988	0.000		0.000
	N	538	529	534	538	538	538	538
IZZ	Spearman Correlation	−**0.147**^**a**^	0.074	**0.169**^**b**^	0.017	−0.031	−**0.441**^**a**^	1
	significance (bilateral)	0.001	0.088	0.000	0.694	0.473	0.000	
	N	538	529	534	538	538	538	538

^a^Correlation is significant at 0.01 (bilateral)

^b^Correlation is significant at 0.05 (bilateral)

*PIU* Problematic Internet Use scored, *BMI* Body Mass Index score, *EAT-26* Eating Attitudes Test score, *BDI* Beck Depression Inventory score, *IZZ* Health-Related Behavior Inventory score

**Table 4:** Comparison of PIU risk groups in terms of the investigated variables (categorical variable)

Variables^a^	*df*	χ^2^	*p* value
Age	3	1.57	NS
Amount of leisure time per day	3	17.65	**< *0.001***
Year of study	3	0.99	NS
BMI	3	2.18	NS
IZZ	3	16.63	**< *0.001***
BDI	3	55.17	**< *0.001***
EAT-26	3	13.93	**< *0.01***

^a^Kruskal–Wallis test for groups at a very low, low, average, high risk of PIU

*BMI* Body Mass Index score, *IZZ* Health-Related Behavior Inventory score, *BDI* Beck Depression Inventory score, *EAT-26* Eating Attitudes Test score, *NS* not statistically significant

### Health behaviors and PIU scores

There were significant, but weak correlations between PIU (continuous variable) and IZZ scores (Table 3). The highest IZZ scores (continuous variable) were, therefore, observed in persons at a very low risk of PIU (categorical variable) *(p* < *0.001)* (Table 4)*.* As shown in Table 2, the IZZ categories significantly differentiated PIU scores (continuous variable) *(p* < *0.05)*. The post hoc tests (Mann–Whitney tests) indicated that students from the low IZZ group scored significantly higher on PIU (continuous variable) than those from the high IZZ group. No PIU differences were observed between the medium IZZ and other groups.

To conduct a more profound analysis of the relationship between PIU and health behaviors, we examined the relationship between IZZ subcategories (healthy eating habits, preventive behaviors, positive psychological attitude, pro-health practices) and PIU score (continuous variable). A significant correlation was demonstrated between the PIU score and the following IZZ subcategories: healthy eating habits (*r* = −0.17, *p* < 0.001), preventive behaviors (*r* = −0.14, *p* < 0.001), and positive psychological attitude (*r* = −0.17, *p* < 0.001). The only sub-category that was not significantly correlated to PIU were health practices. Moreover, persons at a very low risk of PIU (categorical variable) achieved the highest scores in healthy eating habits (*H*_3449_ = 17.09; *p* < 0.001), preventive behaviors (*H*_3449_ = 11.56; *p* < 0.001) and positive mental attitude (*H*_3449_ = 17.09; *p* < 0.001), followed by those at a low and medium PIU risk. Those at the highest risk of PIU (categorical variable) had the lowest scores in the indicated IZZ domains.

### BMI and PIU scores

There was a significant, but weak, correlation between BMI and PIU scores (continuous variable) (Table 3). However, BMI did not differentiate the groups of varying PIU risks (categorical variable) (Table 4).

### Depression and PIU scores

*The strongest* significant correlation was demonstrated between PIU (continuous variable) and BDI scores (*r* = 0.325; *p* < 0.001) (Table 3). The lowest BDI scores were reported by persons at a very low risk of PIU (categorical variable), followed by low-, medium-, and high-risk groups *p* < *0.001* (Table 4). Depression level (the BDI score) significantly differentiated overall PIU scores (continuous variable) *p* < *0.001* (Table 2). Students meeting depression criteria obtained higher overall PIU scores.

### Eating disorders and PIU scores

There were significant associations between PIU (continuous variable) and EAT scores (Table 3). The lowest EAT scores were recorded among persons at a very low risk of PIU (categorical variable), *p* < *0.01* (Table 4)*.* The EAT score of ≥ 20 points did not significantly affect the PIU score (continuous variable) *p* > *0.05* (Table 2)*.*

## Discussion

PIU is associated with symptoms of depression, higher EAT scores, and reduced rigorousness of health-promoting behaviors, such as eating habits, preventive behaviors, or positive mental attitude. There was a negative, though weak correlation between PIU and BMI. It was more common in male, full-time students, who had more free time during the day, used the Internet for entertainment purposes, mostly on a computer rather than a mobile phone.

Our results, i.e., 2.8% of students at a high risk of PIU, indicate that the investigated phenomenon is in fact not as common among health-related majors, as suggested by research conducted among adolescents and students of different majors in Turkey [37], Germany [38], or China [39] (prevalence of approximately 8%). It can be assumed that students of medical and health-related majors may manifest greater ease of establishing, maintaining and using interpersonal contact with other people than the general population. Of note, the tested sample consisted largely of women (83.5%), who are at a lesser risk of PIU than men.

In our study, persons reporting the highest intensity of health behaviors in general and in the individual domains, i.e., eating habits, preventive behaviors or positive mental attitude, were at a low risk of PIU. We expect individuals affected by PIU to lead a less healthy lifestyle due to a greater involvement in Internet use. Previous studies link PIU to low conscientiousness [3]. Less conscientious individuals may be less prone to engage in health behaviors, such as regular physical activity, healthy diet or avoidance of fast- or processed foods, which often require time and effort. We may, therefore, assume that they choose to use the Internet at the expense of observing the principles of a healthy lifestyle.

A significant number of studies suggest links between symptoms of depression and PIU [4–7]. A similar tendency was also observed in this study. The association between depressive symptoms and PIU proved to be the strongest of all the examined variables. Unfortunately, our research model does not allow to draw conclusions about any cause-and-effect relationships between the variables. For this reason, the interpretation of our data can be carried out in two ways. Namely, excessive Internet use may be a part of a process, which in some cases leads to low mood and even onset of a depressive episode. On the other hand, an increased PIU risk can be a response to the ongoing low mood or depression, the causes of which may not be directly related to this phenomenon.

The relationship between PIU and eating disorders has not been studied as thoroughly as the relationship between PIU and depression. Reports indicate a significant correlation between PIU and eating disorders [12, 16–18, 20]. A similar correlation has also been demonstrated in this study, although the strength thereof was rather low.

Our results suggest that PIU is not a common phenomenon among Polish medical school students, who seem to be more likely to suffer from depressive rather than PIU symptoms. Nevertheless, given the widespread digitization of life related to the SARS-CoV-2 pandemic, this issue should still be investigated. Our pre-pandemic data shows that students with a higher PIU score are more likely to lead an unhealthy lifestyle and manifest symptoms of depression and eating disorders. Lifestyle changes caused by a more frequent use of a computer may lead to serious consequences, e.g., an increased risk of developing numerous civilization diseases. This, in turn, can become a serious challenge for public health. Modern trends in health promotion should, therefore, focus more strongly on the problematic use of the Internet. Our research suggests that among health-related majors, PIU prevention should mainly concern male, full-time students who have a lot of free time during the day, use the Internet for entertainment, communication and social media, mostly on a computer or computer and smartphone comparably. Due to the observed prevalence of depressive symptoms in this population, provision of access to psychological and psychiatric care should be considered for medical school students.

### Limitations

Our research was not free of limitations. Since it was a cross-sectional study, we did not have the possibility to clinically confirm either depressive or eating disorders in our sample. The psychometric properties of the IZZ questionnaire should be emphasized. While Cronbach's alpha for the entire scale was 0.85, which is a good result, the values for its four subscales remained in the range from merely 0.60–0.65. In the relationship between PIU and health behaviors, gender could be a confounding variable. The majority of the sample were women, and it is women who more frequently engage in health behaviors and are at a lower risk of PIU. Due to the high homogeneity of the studied sample (education path, sex), the obtained conclusions refer to students of medical universities and should be transferred to the general population with great caution.

## Conclusions

Problematic Internet Use among medical school students is associated with symptoms of depression and eating disorders as well as unhealthy lifestyle. Problematic Internet Use prevention should mainly concern male, full-time students who have a lot of free time during the day, use the Internet for entertainment, communication and social media. Due to the observed prevalence of depressive symptoms in this population, provision of access to psychological and psychiatric care should be considered for medical school students.

## Data Availability

The data sets used and/or analysed during the current study are available from the corresponding author on reasonable request.

## Abbreviations

BDI: The Beck Depression Inventory
EAT-26: The Polish version of the Eating Attitudes Test
IZZ: Health-Related Behavior Inventory
PIU: Problematic Internet Use
PMU: Pomeranian Medical University in Szczecin
TPUI22: The Polish version of Problematic Internet Use Test
